# Retraction: PPARGC1A regulates transcriptional control of mitochondrial biogenesis in early bovine embryos

**DOI:** 10.3389/fcell.2026.2026356

**Published:** 2026-10-22

**Authors:** Frontiers Editorial Office

The journal retracts the 17 January 2025 article cited above.

Following publication, concerns were raised regarding the integrity of the images in the published figures. The authors failed to provide a satisfactory explanation during the investigation, which was conducted in accordance with Frontiers’ policies.

This retraction was approved by the Chief Executive Editor of Frontiers. The authors received a communication regarding the retraction and had a chance to respond. This communication has been recorded by the publisher.

Frontiers would like to thank Dr. Elisabeth Bik for contacting the journal regarding the published article.

